
JOURNAL INFORMATION
==============================
NLM Title Abbreviation: ScientificWorldJournal
Journal ID: ScientificWorldJournal
NLM Journal ID: 101131163
Journal ID: TSWJ

The Scientific World Journal

ISSN: 2356-6140
EISSN: 1537-744X
Publisher: Wiley

ARTICLE INFORMATION
==============================
PMCID: PMC5956450
PMID: 15349521
DOI: 10.1100/tsw.2004.38
Article ID: 182649
Article version: 1
Subjects: Original Report

Intratesticular Adenomatoid Tumor Radiographically Mimicking A Primary Testicular Malignancy

Adler Howard L. 1
Haddad John L. 2
Wheeler Thomas M. 1 3
Kim Edward D. 1
1 Scott Department of Urology, The Methodist Hospital, Baylor College of Medicine, Houston, TX, USA
2 Department of Radiology, The Methodist Hospital, Baylor College of Medicine, Houston, TX, USA
3 Department of Pathology, The Methodist Hospital, Baylor College of Medicine, Houston, TX, USA
Academic Editor: Anthony Atala

Publication date: 2004
Electronic publication date: 2004 Jun 7
Volume: 4
First page: 11
Last page: 15
Copyright: Copyright © 2004 Howard L. Adler et al.
Copyright year: 2004
License: This is an open access article distributed under the Creative Commons Attribution License, which permits unrestricted use, distribution, and reproduction in any medium, provided the original work is properly cited.
License URL: https://creativecommons.org/licenses/by/3.0/

==============================
Original Report
*TheScientificWorldJOURNAL* (2004) 4 (S1), 11–15
ISSN 1537-744X; DOI 10.1100/tsw.2004.38

### Intratesticular Adenomatoid Tumor Radiographically Mimicking A Primary Testicular Malignancy

Howard L. Adler, M.D.[1], John L. Haddad, M.D.[2], Thomas M. Wheeler, M.D.[1,3], and Edward D. Kim, M.D.[1]

*[1]Scott Department of Urology, [2]Department of Radiology, [3]Department of Pathology, The Methodist Hospital, Baylor College of Medicine, Houston, Texas*

*Previously published in the Digital Urology Journal*

**DOMAIN**: urology

### INTRODUCTION

Adenomatoid tumors are benign neoplasms which commonly occur in the paratesticular tissues. Testicular adenomatoid tumors have been reported previously and almost always arise from the tunica albuginea. To date, only three cases of primary intratesticular adenomatoid tumors have been described.[1-3] We now describe a case of a young man who was found to have an intratesticular adenomatoid tumor in continuity with the rete testis.

### CASE HISTORY

A 36-year-old male of Hispanic and African-American descent presented with a three week history of a non-tender right scrotal/testicular lesion. The patient denied any history of prior trauma, epididymitis, cryptorchidism, groin surgery, or unprotected intercourse. On physical examination, the patient was found to have minimal induration at the superior aspect of the right epididymis. The right and left testes were both normal to palpation. Scrotal ultrasound revealed a normal right testicle and normal epididymides. A 6 mm heterogeneous mass was incidentally discovered in the lower pole of the left testicle (Figure 1). Magnetic resonance scan (MRI) demonstrated the lesion to be hypointense to the normal testicular parenchyma on T2-weighted images (Figure 2A). This lesion enhanced greater than the surrounding parenchyma on T1-weighted images obtained after intravenous administration of gadolinium-DTPA (Figure 2B). Serum beta-human chorionic gonadotrophin and alpha-fetoprotein levels were both undetectable. A left radical orchiectomy was performed. Pathology revealed an intratesticular adenomatoid tumor in continuity with the rete testis (Figures 3A and 3B). There was no association of the tumor with the tunica albuginea identified.

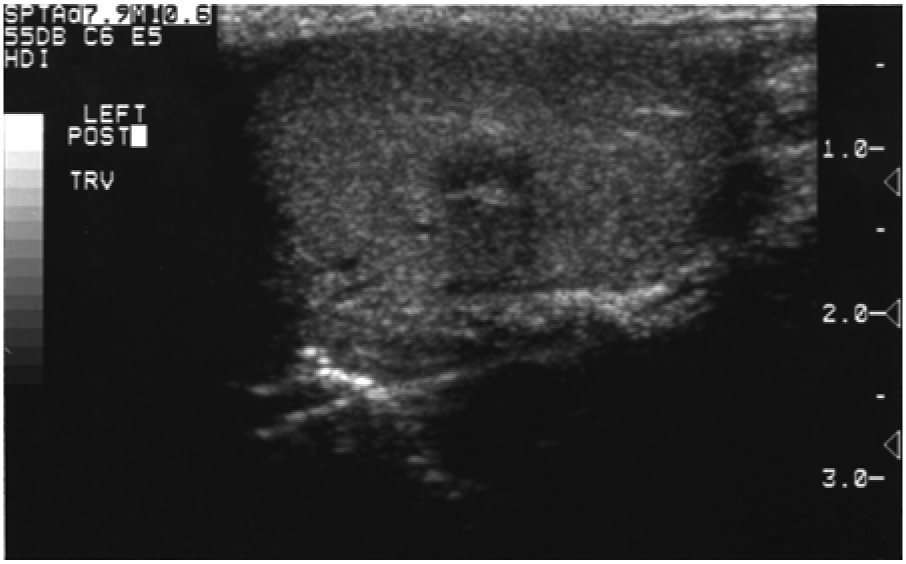

**FIGURE 1**. Scrotal ultrasound identifies a heterogeneous 6 mm mass in the lower pole of the left testicle.

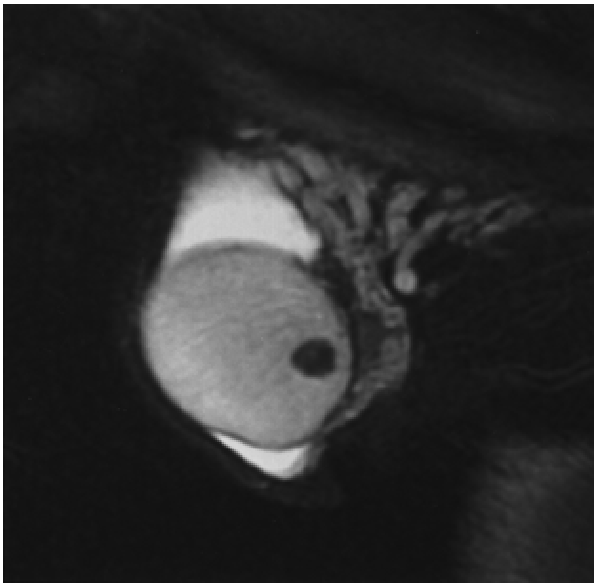

**FIGURE 2A.** The lesion demonstrates low signal intensity on T2-weighted images.

### DISCUSSION

Adenomatoid tumors are the most common tumor of the paratesticular tissues and usually occur between the third and fourth decades of life.[3] Adenomatoid tumors involving the testicle usually arise from the tunica albuginea and invade the testicular parenchyma. Tammela et al describe 8 patients who were found to have adenomatoid tumors of the tunica albuginea. In each case, the tumor was located superficially in the tunica albuginea at the lower pole of the testis. Only one patient had an orchiectomy, while the other patients were treated with local excision. These patients were found to have tumors which were isoechoic, hypoechoic, or heteroechoic by ultrasonography. Furthermore, the lesions were noted to be clearly outside the testicular parenchyma and unlikely to be malignant.[4]

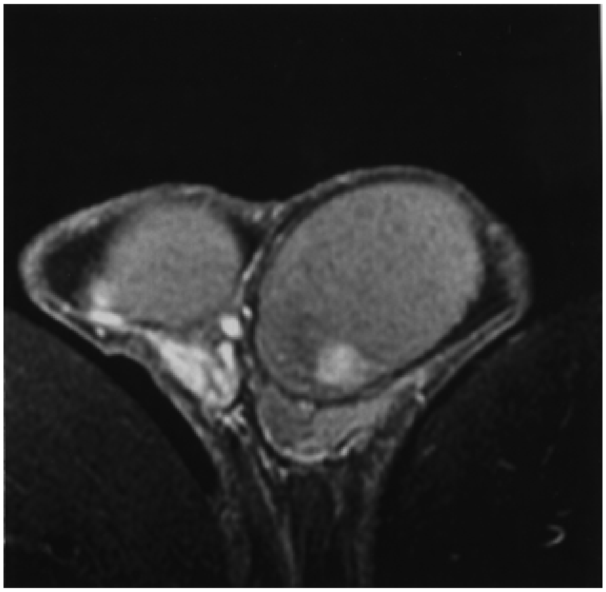

**FIGURE 2B.** There is enhancement greater than the surrounding parenchyma on T1-weighted images post gadolinium-DTPA administration.

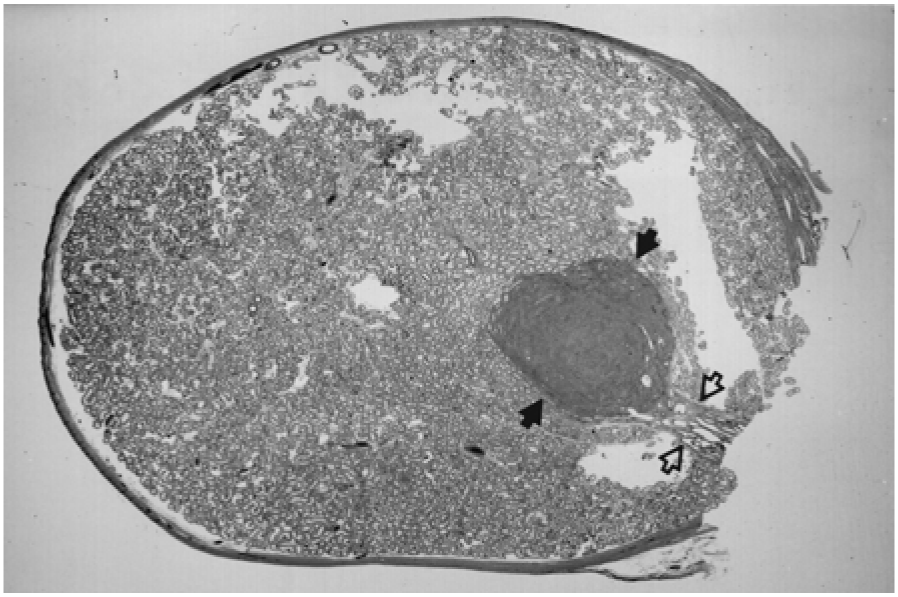

**FIGURE 3A.** Whole mount section of the testis showing the tumor (bold arrows) in continuity with the straight ducts of the rete testis (open arrows). (Hematoxylin and eosin, original magnification X 1).

In contrast, three cases of primary intratesticular adenomatoid tumors have been previously described.[1-3] In each case, the benign nature of the testicular mass could not be determined preoperatively. Two of the three previous cases were treated with inguinal orchiectomy.[1,2] Moreover, the patient described by Somers was found to have an intratesticular lesion by ultrasonography.[2] The patient described by Samad et al refused orchiectomy unless a malignancy could be identified; consequently, he had primary excision of the lesion with intraoperative frozen sections.[3]

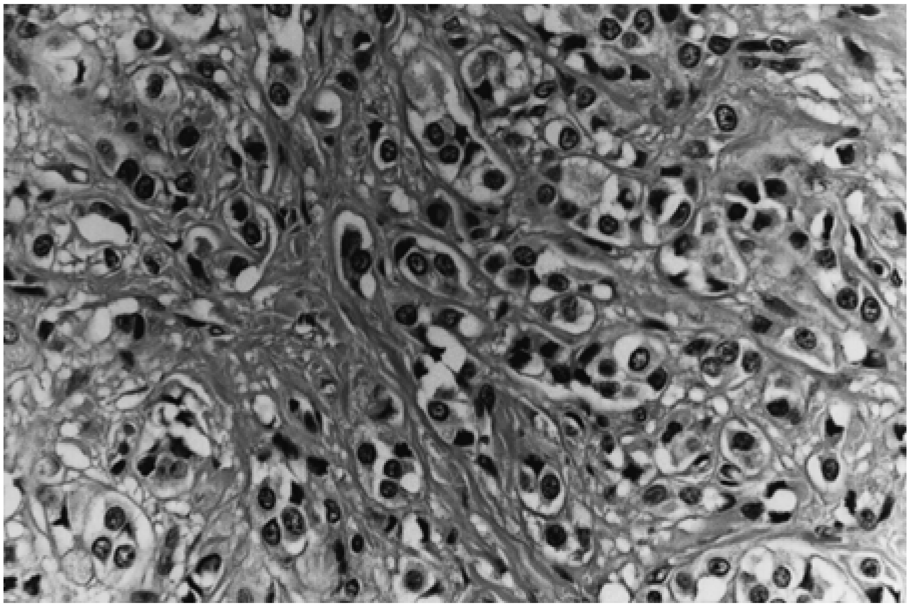

**FIGURE 3B.** Photomicrograph of a representative section of the tumor showing bland cellular structures in a dense fibrous background. (Hematoxylin and eosin, original magnification X 400).

Our patient had a heterogeneous mass of the left testicle identified by ultrasonography. Feuer et al described the ultrasonographic characteristics of three men with testicular adenomatoid tumors. Two patients had isoechoic lesions while the third patient had a normal testicular ultrasound. The authors, however, did not state if these lesions were contiguous with the tunica albuginea.[5] These findings are in contrast to those of the suspicious ultrasonographic findings in our patient and in the patient described by Somers.[2]

The MRI appearance of this lesion has not been previously reported. MRI scan only served to heighten our suspicion that this was a malignant lesion, as the presence of a discrete solid enhancing lesion was confirmed. Our case is also distinguished from the other reported cases in that this tumor was identified on diagnostic ultrasonography for symptomatic complaints in the opposite testicle. Scrotal ultrasonography and MRI failed to identify any pathology within the right testicle.

In conclusion, testicular adenomatoid tumors are rare benign lesions with clinical presentations similar to that of malignant testicular neoplasms. The radiographic findings of testicular adenomatoid tumors are variable. Patients who present with vague scrotal and/or testicular complaints may benefit from scrotal imaging studies. Furthermore, any suspicious lesions identified by sonography should be treated with inguinal orchiectomy given the extremely low incidence of benign testicular neoplasms. Patients with isoechoic or sonographically normal lesions may benefit from inguinal exploration with excisional biopsy and frozen sections.

### REFERENCES

1. Horstman, W.G., Sands, J.P. and Hooper DG: Adenomatoid tumor of the testicle. Urology 40: 359, 1992.
2. Somers, W.J.: The sonographic appearance of an intratesticular adenomatoid tumor. J Clin Ultrasound 20: 479, 1992.
3. Samad, A.A., Pereiro, B., Badiola, A., Gallego, C. and Zungri, E.: Adenomatoid tumor of intratesticular localization. Eur Urol 30: 127, 1996.
4. Tammela, T.L.J., Karttunen, T.J., Makarainen, H.P., Hellstrom, P.A., Mattila, S.I. and Konturri, M.J.: Intrascrotal adenomatoid tumors. J Urol 146: 61, 1991.
5. Feuer, A., Dewire, D.M. and Foley, W.D.: Ultrasonographic characteristics of testicular adenomatoid tumors. J Urol 155: 174, 1996.

**This article should be referenced as follows:**

Adler, H.L., Haddad, J.L. Wheeler, T.M., and Kim, E.D. (2004) Intratesticular adenomatoid tumor radiographically mimicking a primary testicular malignancy. *TheScientificWorldJOURNAL* **4 (S1)**, 11–15.

**Handling Editor:**

Anthony Atala, Principle Editor for *Urology* — a domain of *TheScientificWorldJOURNAL*.
